# Identification of Blood Meal Sources in *Aedes vexans* and *Culex quinquefasciatus* in Bernalillo County, New Mexico

**DOI:** 10.1673/031.013.7501

**Published:** 2013-08-05

**Authors:** Jacob A. Greenberg, Daniel A. Lujan, Mark A. DiMenna, Helen J. Wearing, Bruce V. Hofkin

**Affiliations:** 1 Department of Biology, University of New Mexico, 167 Castetter Hall MSC03 2020, Albuquerque, New Mexico 87131-0001; 2 Urban Biology Division, City of Albuquerque Environmental Health Department, P.O. Box 1293, Albuquerque, New Mexico 87103

**Keywords:** host feeding, mosquito, West Nile virus, arbovirus

## Abstract

*Culex quinquefasciatus* Say (Diptera: Culicidae) and *Aedes vexans* Meigen are two of the most abundant mosquitoes in Bernalillo County, New Mexico, USA. In this study, a polymerase chain reaction based methodology was used to identify the sources of blood meals taken by these two species. *Ae. vexans* was found to take a large proportion of its meals from mammals. Although less specific in terms of its blood meal preferences, *Cx. quinquefasciatus* was found to feed more commonly on birds. The results for *Ae. vexans* are similar to those reported for this species in other parts of their geographic range. *Cx. quinquefasciatus* appears to be more variable in terms of its host feeding under different environmental or seasonal circumstances. The implications of these results for arbovirus transmission are discussed.

## Introduction

Mosquito blood meal identification has been used to better understand the feeding behaviors of various mosquito species in many areas across the United States and the world (Irby and Apperson 1988; Zinser et al. 2004; Gingrich and Williams 2005; Molaei and Andreadis 2006; Molaei et al. 2006, 2007; Kay et al. 2007; Garcia-Rejon et al. 2010; Sawabe et al. 2010; Barrera et al. 2011). Here, the results of a similar study, using a polymerase chain reaction (PCR) based methodology designed to assess the host feeding patterns of *Aedes vexans* Meigen (Diptera: Culicidae) and *Culex quinquefasciatus* Say in Bernalillo County, New Mexico, USA, are presented

*Ae. vexans* and *Cx. quinquefasciatus* are two of the most abundant mosquito species in Bernalillo County (DiMenna et al. 2006). *Ae. vexans* mosquitoes are known to feed aggressively on mammals, including humans (Tempelis et al. 1965; Cupp and Stokes 1973; Magnarelli 1977a, b; Ritchie and Rowley 1981; Nasci 1984; Irby and Apperson 1988; Apperson et al. 2002, 2004; Lee et al. 2002; Hassan et al. 2003; Gingrich and Williams 2005; Molaei et al. 2006). *Cx. quinquefasciatus*, alternatively, is less specific in its feeding, but typically takes a majority of its blood meals from avian hosts. (Bohart et al. 1978; Kilpatrick et al. 2006; Savage et al. 2007; Garcia-Rejon et al. 2010). In some cases, *Cx. quinquefasciatus* feeds equally on both birds and mammals, or can show a mammalian bias. (Zinser et al. 2004; Kay et al. 2007; Molaei et al. 2007; Muturi et al. 2008; Swabe et al. 2010).

Both of these mosquito species are known arbovirus vectors of medical and veterinary importance. *Ae. vexans* is known to transmit St. Louis encephalitis and Western Equine Encephalitis, and is a potential bridge vector for West Nile virus (WNV) (Turell et al. 2001; Cupp et al. 2004; Kilpatrick et al. 2005; DiMenna et al. 2006; Molaei and Andreadis 2006; Blitvich 2008). Likewise, *Cx. quinquefasciatus* is known to be a highly competent vector for WNV in North America, and because of its strong tendency to feed on birds, it may play a particularly important role in viral amplification. (Andreadis et al. 2001; Bernard et al. 2001; Kulasekera et al. 2001; Nasci et al. 2001; Sardelis et al. 2001; Turell et al. 2001; White et al. 2001; Goddard et al. 2002; Turell et al. 2002, 2005; Anderson et al. 2004; Andreadis et al. 2004; Solomon 2004; Ebel et al. 2005; Kilpatrick et al. 2005; Hayes and Gubler 2006). Both of these mosquito species, along with a third species, *Culex tarsalis*, have tested positive for WNV infection in Bernalillo County (DiMenna et al. 2006).

Blood meal source, along with other aspects of mosquito biology such as biting frequency, dispersal ability, and local abundance, are important components of vector capacity (Seagerman et al. 2008; Chaves et al. 2010). A greater understanding of host utilization by mosquitoes in Bernalillo County can help clarify the dynamics of local arbovirus transmission, specifically the amplification cycle of vector-borne viruses in their natural hosts, and their transmission to humans and other domestic animals. Such data might consequently lead to more effective and focal vector control.

## Materials and Methods

Bernalillo County, in central New Mexico, includes the greater Albuquerque metropolitan area, which accounts for 32.5% of the state's population (City of Albuquerque 2010). The Rio Grande flows directly through the county from north to south, forming the Rio Grande Valley. Much of the valley retains a rural character. The riverbanks are generally heavily wooded and form a riparian forest known as the Rio Grande Bosque. Outside of Albuquerque, the lands adjacent to the Bosque are often devoted to agriculture or grazing. Most mosquito activity, and consequently most arbovirus transmission in Bernalillo County, occurs in the Rio Grande Valley (DiMenna et al. 2007). Over 50 mosquito species have been collected in New Mexico (Wolff and Nielsen 2007). This study focuses on *Cx. quinquefasciatus* and *Ae. vexans*, two of the most common species in central New Mexico.

**Figure 1.**
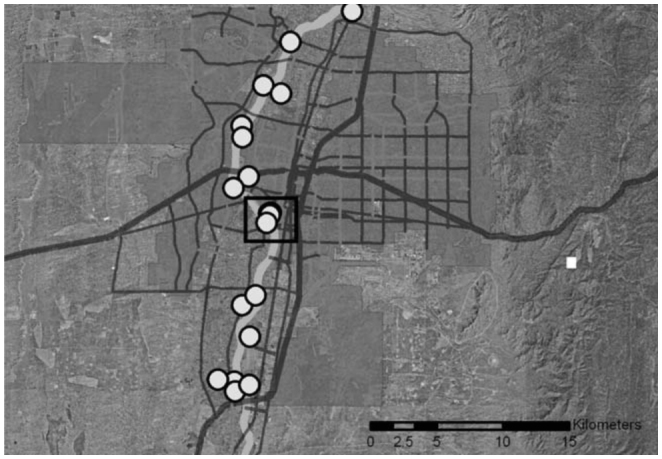
Mosquito trapping sites along the Rio Grande Bosque in Bernalillo County are marked with white circles. The light gray line from north to south represents the Rio Grande. The box indicates trap sites located at the Rio Grande Zoo. High quality figures are available online.

### Mosquito collection, identification, and WNV screening

Mosquitoes were collected weekly throughout the mosquito season (May through October) from 2006 through 2010. Each year, between 18 and 22 trapping sites were established along the Rio Grande Bosque in Bernalillo County (Figure 1). Two types of mosquito trap were used at each site. CDC light traps were suspended 1.5 m from the ground and baited with a thermos canister containing approximately 1.5 kg of dry ice, and gravid traps were baited with non-chlorinated water infused with horse manure, grass clippings, and bacterial culture (Pro-pump Liquid Live Bacteria High Count, Ecological Laboratories, www.microbelift.com), which was allowed to ferment for two weeks. Traps were set in the late afternoon, left overnight, and collected the following morning. Collected mosquitoes were immediately placed on dry ice and were subsequently stored at -80° C. Mosquitoes were identified to species using dichotomous keys (Carpenter and Lacasse 1955; Pratt and Barnes 1959; Darsie and Ward 1981). Blood-engorged mosquitoes were set aside for blood meal analysis. Mosquitoes not clearly engorged with a blood meal were tested for WNV as described by Lanciotti et al. (1999). Maximum likelihood estimate of infection and their 95% confidence intervals were determined using the Biggerstaff (2007) Add-in for Microsoft Excel (www.microsoft.com). Relative abundance of each mosquito species was calculated by dividing the number of each species collected by the combined total of collected mosquitoes for each collection season.

### Blood meal analysis

Blood-engorged mosquitoes were placed individually on a microscope slide under a dissecting microscope. The midgut and abdomen were removed using a razor blade and sterile forceps. A new slide and blade were used for each mosquito. Genomic DNA was then extracted from each midgut and abdomen with a modified DNAzol BD (Molecular Research Center, www.mrcgene.com) procedure as previously described by Molaei et al. (2006).

The source of each blood meal was determined by subjecting each sample of genomic DNA to two separate PCR reactions, one to identify mammalian and one to identify avian DNA. Mammalian blood meals were identified using mammalian-specific primer pairs for a 772 bp portion of the *cytochrome b* gene (Ngo and Kramer 2003). Likewise, avian blood meals were identified by amplifying a 508 bp fragment of the *cytochrome b* gene with the avian-specific primers (Cisneros and Johnson 2001). Each 50 µl reaction contained 300–400 ng of genomic DNA serving as template, 5 µl of 10x buffer (Roche Applied Science, www.roche-applied-science.com), 8µl dNTPs (200 µM of each; Applied Biosystems, www.invitrogen.com), 8 µL MgCl_2_ (4 mM; Roche Applied Science), 5 µl forward primer (0.5 µM), 5 µl reverse primer (0.5 µM), and 0.25 µl TaqGold Polymerase (1.25 U per reaction; Roche Applied Science). Sterile water (Sigma-Aldrich, www.sigmaaldrich.com) was added to bring the total reaction volume to 50 µl. Primer sequences and cycling conditions have been previously published by Greenberg et al. (2012). A negative water control lacking template was included with all PCR reactions. A second negative control consisted of a DNAzol extract lacking mosquito midgut. All reactions also included a positive control containing either mammalian (*Mus musculus*) or avian (*Zenaida macroura*) genomic DNA serving as a template. If a sample did not amplify, a second PCR reaction was performed. After two failed amplifications, the sample was archived in a -80° C freezer.

Amplified PCR products were purified with one of several methods, including a size select e gel (Invitrogen, www.invitrogen.com), PCR purification kit (Qiagen, www.qiagen.com), minielute column (Millipore,www.millipore.com), or exo-sap (Affymetrix, www.affymetrix.com). Amplicons were directly sequenced with a Big Dye 3.1 sequencing kit, using the big dye step protocol PCR regime (Platt et al. 2007). Samples were then sequenced on an ABI 3130 DNA Sequencer (Applied Biosystems, Foster City, CA, USA) at the University of New Mexico, Department of Biology Molecular Facility. Sequences were edited using Sequencher version 4.10.1 (Gene Codes, www.genecodes.com) and identified to species through a BLAST search comparison with the GenBank DNA database (www.ncbi.nlm.nih.gov/blast/Blast.cgi). Those comparisons with a blast error value < le^-20^ were included in our analysis.

### Statistical analysis

To determine if each of the two mosquito species under investigation were more likely to feed on either mammalian or avian hosts, all successfully identified blood meals for each species were scored as either “mammalian” or “avian” and subjected to a z-test to determine significant deviation from 50/50 mammalian/avian feeding for the mosquitoes collected in this study. Confidence intervals of 95% were found for each species' tendency to feed on either mammalian or avian hosts, and the species were compared for overlap. Calculations with non-overlapping 95% confidence intervals and found to be two standard deviations from the null hypothesis of no deviation were considered to be significant.

## Results

**Figure 2.**
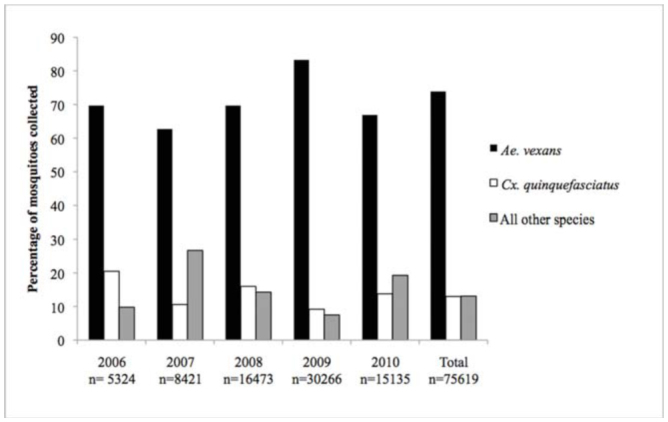
Mosquito abundance in Bernalillo County. The number of *Aedes vexans, Culex quinquefasciatus*, or all other species combined was divided by the total number of identified mosquitoes to determine the proportion of trapped mosquitoes belonging to each species. High quality figures are available online.

A total of 75,619 mosquitoes from 24 species were collected from 2006–2010. Of these mosquitoes, 55,871 (73.9% of total) were *Ae. vexans*, while 9,853 (13.0% of total) were *Cx. quinquefasciatus* (Figure 2). These two species accounted for 65,724 (86.9%) of the total number of mosquitoes collected. The maximum likelihood estimate of infection of *Ae. vexans* and *Cx. quinquefasciatus* were found to be .05/1000 (95% confidence interval of 0.00-0.25) and 1.77/1000 (95% confidence interval of 0.97–3.01) respectively.

**Figure 3.**
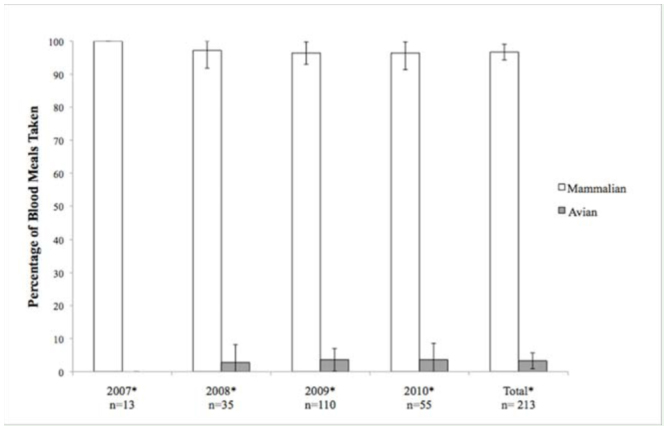
Yearly and total feeding patterns of *Aedes vexans*. (*) denotes statistical significance from the null hypothesis of 50/50 feeding pattern Error bars indicate 95% confidence interval. The number of SD from the null hypothesis was found to be 13.6. High quality figures are available online.

**Figure 4.**
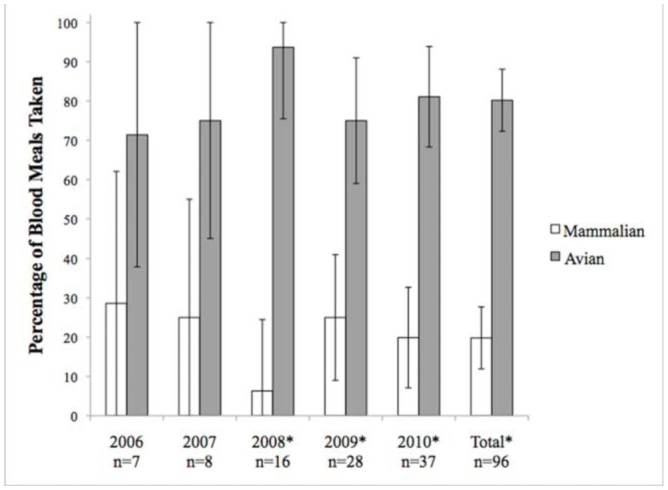
Yearly and total feeding patterns of *Culex quinquefasciatus*. (*) denotes statistical significance from the null hypothesis of 50/50 feeding pattern. Error bars indicate 95% confidence interval. The number of SD from the null hypothesis for the total was found to be 5.9. High quality figures are available online.

A total of 309 out of 337 amplified blood meals (91.7%) were successfully identified to species of origin (Tables 1, 2). Of these, 37 (11.2%) were identified as exotic animals housed in the Rio Grande Zoo. *Ae. vexans* consistently fed largely on mammalian hosts, most commonly cows, horses, and cottontail rabbits (Figure 3). Of the 213 successfully identified blood meals from this species, 206 (96.7 ± 2.4%) were identified as mammalian in origin, while 7 (3.3 ± 2.4 %) were avian. The majority of identified *Cx. quinquefasciatus* blood meals were consistently taken from avian hosts, the most common of which were American robins, house sparrows, and mourning doves (Figure 4). Of the 96 successfully identified blood meals, 77 (80.2 ± 7.9%) were avian in origin, while 19 (19.8 ± 7.9%) were mammalian. No mixed mammalian and avian meals were identified for either species.

## Discussion

In this study, a PCR-based method was used to identify the blood meal sources of two of the most common mosquito species in Bernalillo County, New Mexico. The results largely confirm those of Loftin et. al. (1997), who also investigated feeding behavior of mosquitoes in central New Mexico. In this earlier study, in which live mosquitoes were offered a choice of both a mammalian and avian blood meal source, *Ae. vexans* almost invariably fed on the available mammal. *Cx. quinquefasciatus*, on the other hand, took a majority of its meals from the avian host, but was substantially less specific, and took a number of mammalian blood meals as well. Similarly, in our study it was found that *Cx. quinquefasciatus* took over 80% of its blood meals from avian hosts, while over 96% of identified *Ae. vexans* blood meals were identified as mammalian in origin, thus confirming Loftin's lab-reared mosquito results with field-collected mosquitoes.

Furthermore, the results are in general agreement with those of others who have considered the feeding behavior of *Ae. vexans* in other geographic areas. Across its geographic range, this species shows a strong tendency to feed on mammals. *Cx. quinquefasciatus*, however, may be more strongly influenced by local or seasonal conditions. In Harris County, Texas, and in Tucson, Arizona, for instance, it was found that *Cx. quinquefasciatus* fed more frequently on mammals (Zinser et al. 2004; Molaei et al. 2007), while in Yucatan, Mexico, this species tended to feed on birds (Garcia-Rejon et al. 2010).

The factors that influence the feeding behavior of *Cx. quinquefasciatus* await elucidation. One possible contributing factor may be the seasonal shift in hosts that some species, including *Cx. quinquefasciatus*, have been shown to undergo in other regions. For example, in Harris County, Texas, *Cx. quinquefasciatus* feeds primarily on birds early in the mosquito season. As the season progresses, mammals make up an increasingly larger proportion of their blood meals (Molaei et al. 2007). There are not as yet sufficient data to determine whether or not a similar feeding shift occurs in Bernalillo County. If, however, such a shift is a general feature of *Cx. quinquefasciatus* feeding biology, it may at least in part explain the variable feeding behavior that has been described for this species in different parts of its geographic range.

The data also highlight the fact that neither *Cx. quinquefasciatus* nor *Ae. vexans* are strongly host-specific towards a particular species. *Cx. quinquefasciatus*, which was found to more commonly feed on birds, utilizes a wide range of avian species as sources for blood meals. Likewise, *Ae. vexans*, which fed almost exclusively on mammals, takes blood from a variety of mammalian hosts. Even exotic species housed at the Rio Grande Zoo, where several of the mosquito trapping sites were located, served as blood meal sources. Many of these species are rarely if ever normally encountered in their natural habitat by mosquitoes native to New Mexico. Furthermore, without data on the numbers of potential avian and mammalian hosts in a particular collection area, it is premature to suggest that the results indicate feeding preferences on the part of the mosquitoes that were collected. However, given the widely different feeding patterns observed in mosquitoes from the same collection sites, it seems unlikely that the observed patterns merely reflect host availability. Because *Cx. quinquefasciatus* occasionally feeds on reptiles and amphibians (Savage et al. 2007), at least some of the failed amplifications may reflect the fact that blood meals were of neither mammalian nor avian origin. Such blood meals, however, are likely to be infrequent, and this possibility would not have substantially altered the results.

Because the source of its blood meals is an important component of a particular mosquito species' vector capacity, the results may help to clarify the roles of *Cx. quinquefasciatus* and *Ae. vexans* in arbovirus transmission in central New Mexico. Both of these species have regularly tested positive for WNV in Bernalillo County (DiMenna et al. 2006). Elsewhere in North America, *Culex spp*. mosquitoes, in particular *Cx. quinquefasciatus*, *Cx. restuans*, *Cx. pipiens*, and *Cx. tarsalis*, have been implicated as the most important WNV vectors (Andreadis et al. 2001; Bernard et al. 2001; Kulasekera et al. 2001; Nasci et al.2001; Sardelis et al. 2001; Turell et al. 2001; White et al. 2001; Goddard et al. 2002; Turell 2002, 2005; Anderson et al. 2004; Andreadis et al. 2004; Ebel et al. 2005; Kilpatrick et al. 2005). Turell et al. (2005) have also demonstrated WNV vector competence for *Ae. vexans*. Because *Ae. vexans* shows such a strong tendency to feed on mammals, it may play an especially important role in bridge transmission to mammals, including humans and horses. It is important to note, however, that *Ae. vexans* is only very rarely found to be infected with WNV in local collections, making its role in WNV transmission questionable. Alternatively, *Cx. quinquefasciatus*, with a significant tendency to feed on birds, may be especially important in viral amplification in avian reservoir hosts such as the American robin (*Turdis migratorius*) (Hamer et al. 2009). A third mosquito species, *Cx. tarsalis*, has also tested positive for WNV in Bernalillo County (DiMenna et al. 2006). Because *Cx. tarsalis* is relatively uncommon at the trapping sites in our study, there are correspondingly few identified blood meals from this species. Determination of this species' host preference awaits further results.

If the findings regarding the feeding behavior of *Cx. quinquefasciatus* and *Ae. vexans*, along with similar data for *Cx. tarsalis*, can be considered with other aspects of vector capacity, it may be possible to use such information to more effectively control arbovirus transmission. These results increase the understanding of the vector-host relationship in the Bernalillo County area, and provide a more definitive picture of the dynamics of WNV transmission in this environment. Existing knowledge of seasonal trends in abundance and spatial distribution of these species, as well as active surveillance, could help determine the most effective allocation of mosquito control resources in order to provide the most effective control measures possible. As additional data becomes available, future research will be directed towards determining how blood meal sources may vary throughout the mosquito season in this study area, providing further opportunities to refine mosquito control strategies and improve the understanding of local mosquito ecology.

**Table 1.**
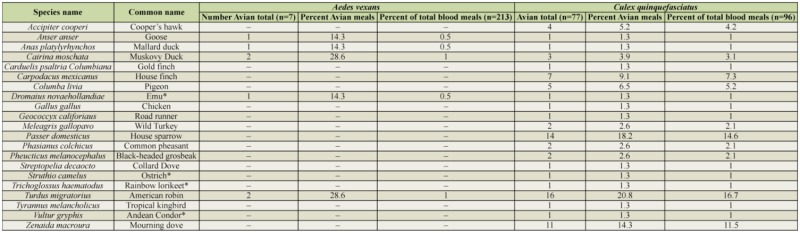
Avian blood meals identified from either *Aedes vexans* or *Culex quinquefasciatus*. Exotic avian species, indicated by (*), were identified from mosquitoes captured at the Rio Grande Zoo.

**Table 2.**
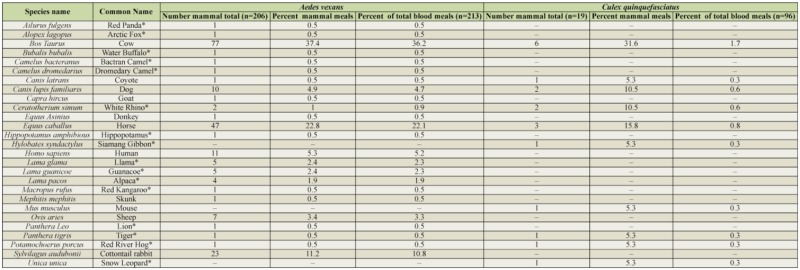
Mammalian blood meals identified from either *Aedes vexans* or *Culex quinquefasciatus*. Exotic mammalian species, indicated by (*), were identified from mosquitoes captured at the Rio Grande Zoo.
